# Primary Prevention from the Epidemiology Perspective: Three Examples from the Practice

**DOI:** 10.1186/1471-2288-10-10

**Published:** 2010-02-03

**Authors:** Iris Pigeot, Stefaan De Henauw, Ronja Foraita, Ingeborg Jahn, Wolfgang Ahrens

**Affiliations:** 1 Bremen Institute for Prevention Research and Social Medicine, University of Bremen, Germany; 2 Department of Public Health, Ghent University, Ghent, Belgium

## Abstract

**Background:**

Primary prevention programmes are of increasing importance to reduce the impact of chronic diseases on the individual, institutional and societal level. However, most initiatives that develop and implement primary prevention programmes are not evaluated with scientific rigor. On the basis of three different projects we discuss necessary steps on the road to evidence-based primary prevention.

**Discussion:**

We first discuss how to identify suitable target groups exploiting sophisticated statistical methods. This is illustrated using data from a health survey conducted in a federal state of Germany. A literature review is the more typical approach to identify target groups that is demonstrated using a European project on the prevention of childhood obesity. In the next step, modifiable risk factors and realistic targets of the intervention have to be specified. These determine the outcome measures that in turn are used for effect evaluation. Both, the target groups and the outcome measures, lay the ground for the study design and the definition of comparison groups as can be seen in our European project. This project also illustrates the development and implementation of a prevention programme. These may require active involvement of participants which can be achieved by participatory approaches taking into account the socio-cultural and living environment. Evaluation is of utmost importance for any intervention to assess structure, process and outcome according to rigid scientific criteria. Different approaches used for this are discussed and illustrated by a methodological project developed within a health promotion programme in a deprived area. Eventually the challenge of transferring an evidence-based intervention into practice and to achieve its sustainability is addressed.

**Summary:**

This article describes a general roadmap to primary prevention comprising (1) the identification of target groups and settings, (2) the identification of modifiable risk factors and endpoints, (3) the development and implementation of an intervention programme, (4) the evaluation of structure, process and outcome and (5) the transfer of an evidence-based intervention into practice.

## Background

Primary prevention programmes are of increasing importance to reduce the impact of chronic diseases on the individual, institutional and societal level. Thus, not surprisingly countless initiatives develop and implement primary prevention programmes for instance in settings as kindergartens and schools to prevent e.g. overweight and obesity in childhood. Typically these programmes are not evaluated with scientific rigor, thus missing an evidence-base for their effectiveness. The evaluation of primary prevention programmes may consider the scientific evidence (external evidence), the motivation and experience of the involved actors (internal evidence) as well as the motivation and problem perception of the target groups. Various classification systems that define hierarchical evidence levels are at hand to assess the degree of scientific evidence according to standardized criteria [see e.g. [1]].

Before addressing the various aspects related to the development, implementation and evaluation of primary prevention programmes let us roughly distinguish primary prevention from health promotion. In our view, primary prevention intends to prevent ill health and targets a well-defined outcome such as a specific health related state like hypertension, obesity, cardiovascular diseases or diabetes. To achieve this primary prevention focuses on the reduction of modifiable risk factors for a given outcome and may thus be considered as somewhat reductionistic. However, since it intends to change the occurrence of specified endpoints it is amenable to a strict outcome evaluation. In contrast, health promotion as a more general approach intends to build up supportive structures, to empower personal resources and to maintain health in a broader sense. It represents a more holistic approach where an epidemiological proof of the effectiveness of a specific health promotion measure in terms of a specific outcome is less suitable.

In the following we will focus on those aspects of prevention and health promotion that are amenable to a stringent evaluation. We will describe a general roadmap to primary prevention where we discuss each step from the identification of target groups up to the transfer into practice. The various steps will be illustrated using three studies that are conducted by the Bremen Institute of Prevention Research and Social Medicine (BIPS), partly in cooperation with international or national partners. These studies are the IDEFICS study (Identification and prevention of dietary- and lifestyle-induced health effects in children and infants), which is an Integrated Project within the 6th Framework Programme of the EU targeting lifestyle- and nutrition-related diseases and disorders among children [2], HEALTH! which is a population-based survey in Bremen addressing the health status among the Bremen population and their access to the health system [3], and Quali-Set-Practice which deals with a participatory evaluation of primary prevention and health promotion in the setting town district of a low socio-economic status. All projects will be described in more detail when they are introduced for illustrative purposes.

## Discussion

### Identification of target groups and settings

The identification of specific subpopulations for targeted interventions is the basic step in course of the development of an effective prevention programme. The criteria for the selection of appropriate target groups involve the degree to which the groups are affected, the feasibility to reach them effectively and the chance to achieve profound and sustainable effects. Furthermore, the aims of the intervention (e.g. reduction of smoking rates) as well as the evidence-base for adequate prevention strategies or even their availability and feasibility (e.g. individual-based vs. setting-based approaches) have to be taken into account. This section presents two different concepts to identify target groups for whom specific prevention measures should be developed.

The usual way for searching the evidence-base relies on a literature review where all available publications have to be screened and assessed regarding their evidence level. Published reviews e.g. by the Cochrane Collaboration on the research field of interest are especially helpful as e.g. those on obesity prevention programmes by Summerbell et al. [4,5], and Stice et al. [6]. When we started to work on the IDEFICS study where the main focus is on the question how to combat the obesity epidemic the results of our literature review could be roughly summarized as: (1) prevention programmes for adults are in general not effective; (2) secondary prevention, i.e. therapy, is often not sustainable [7], and (3) childhood obesity tracks into adult life [8,9]. Since in addition families with low socio-economic status (SES) or migrants are often most affected [10,11] it is of utmost importance to reach the whole population through an obesity prevention programme [12]. This means that programmes to prevent overweight and obesity should start as early in life as possible, i.e. the target group should be children, and should reach vulnerable groups (families of low SES, with migrant background or single parent families) e.g. via the settings kindergarten and school. We therefore decided to focus the IDEFICS study on children in Europe aged 2 to 10 years and to approach them via schools and kindergartens [13,14].

**Figure 1:**
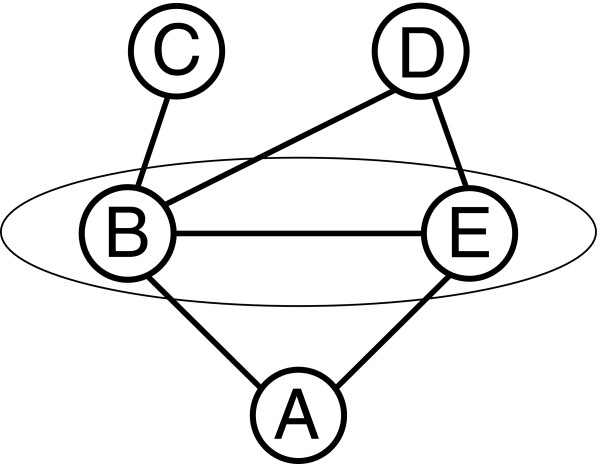
**Graph representing conditional independence of A and the set of variables C and D given the information on B and E**.

Besides the above approach general health surveys or health reporting can reveal needs for prevention and accordingly target groups. Such surveys can help to identify groups with high or low health risks according to (1) factors on a macro level like social inequality, measured e.g. by education, and social position, measured e.g. by employment status, marital status, composition of household, age and place of residence, as well as according to (2) factors on a micro level like health behaviour, measured e.g. by smoking, physical activity, seeking advice, number of medical consultations and participation in screenings. Other factors on the micro level are the self-assessment of one's own health behaviour and the self-efficacy. Pigeot et al. [15] as well as Jahn and Foraita [16] present statistical methods that are able to detect complex association structures where a huge number of variables is involved. These typically exploratory techniques make use of conditional probabilities such as the probability of, say, getting a specific disease conditional on a certain pattern of risk factors. Although these techniques are not such simple in nature their results can be easily communicated by an adequate visualization as graphs or trees. The approach that we present here is based on a combination of two multivariate statistical tools. In a first step, we used so-called graphical models [17,18] where we analyzed the Bremen survey HEALTH! as an example. This technique models and represents conditional probabilities in a graph. Consider for instance five variables denoted as A, B, C, D and E. The fact that A is conditionally independent of C and D given the information on B and E, in terms: A ⊥ {C, D} | {B, E}, can be represented in a graph as depicted in Figure 1. This method allows for reducing the complexity of a data set by selecting the potentially relevant factors to be used afterwards for a more detailed analysis.

That is, in a second step, we applied CHAID-decision trees (Chi-squared Automatic Interaction Detector) [19,20] for the more detailed analysis now based on a reduced set of variables, namely those that have been selected by graphical models. CHAID-decision trees enable the user to detect patterns in huge datasets and to illustrate them in a tree by conditional probabilities.

Both techniques can of course also be used separately but their combination makes them especially useful for our purpose. Whether one method outperforms the other has not yet been investigated. This may be due to the fact that they are typically not used for the same purpose as it is the case in our analysis, too. One should also be aware of the fact that our approach requires a large sample size to achieve a sufficient number of observations in each cell, i.e. for each combination of variables. Otherwise the results may become unstable.

**Figure 2:**
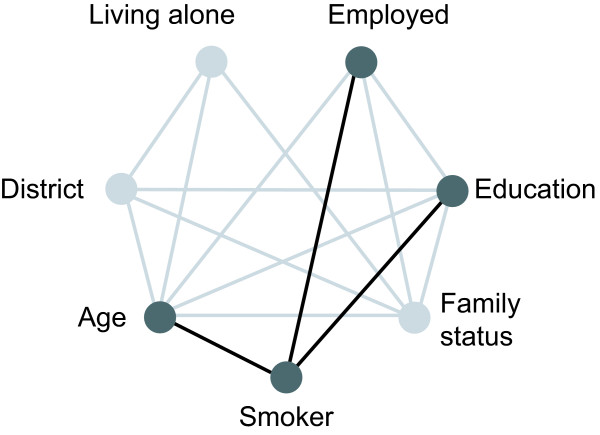
**Independence graph for male smokers**.

Let us illustrate this idea of exploiting statistical methods for the identification of health risks and thus for identifying specific target groups by an example from the Bremen survey HEALTH! This survey was conducted from September to November 2004. A questionnaire and two reminders were sent to a random sample of the Bremen population. In total, 4,891 female inhabitants between 18 and 80 years and 4,647 male inhabitants have been contacted. A response proportion of 43.2% among women and 35.2% among men resulted in data on 2,070 women and 1,521 men. It is well known that smoking is an important risk factor for many diseases. Thus, revealing groups that are especially prone to smoking helps to develop a tailored programme for smoking prevention. We present here the results for male smokers where we only consider variables on the macro level. The independence graph shown in Figure 2 identifies employment status, age and education as associated with the smoking status of men. These variables are entered in a CHAID analysis.

**Figure 3:**
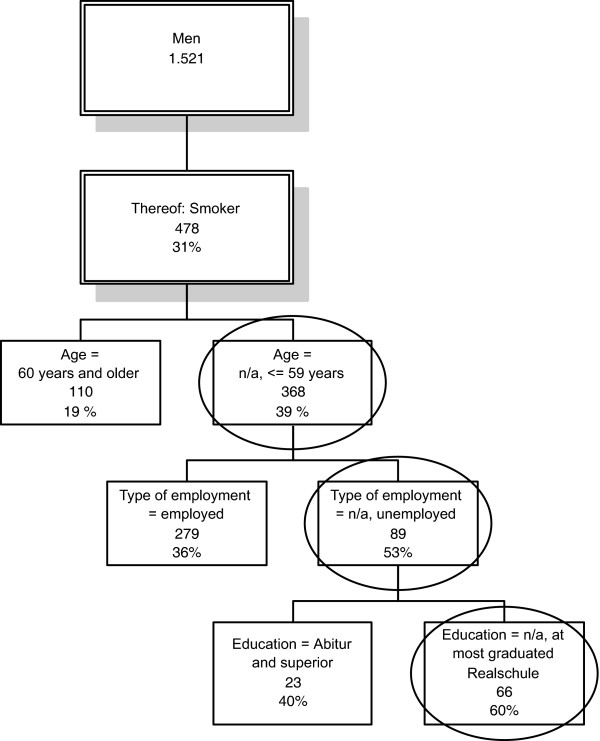
**Decision tree for male smokers (Abitur is a university-entrance diploma [completed 13 years of school]; Realschule corresponds to secondary school level [10 years of school])**.

Starting with 1,521 men of whom 476 are current smokers the automatic search for the most important variable to distinguish this group further gives the variable age. Smoking prevalence is 39% among men under 60 and 19% in men above this age. While no further differentiation is possible in older men, employment status is the next important variable in younger men. Below the age of 60 53% of the unemployed men are smokers as compared to 36% of the employed men. Investigating the unemployed group further, the prevalence of smoking is highest in men, whose highest educational attainment is the German Realschule (type of secondary school) (see Figure 3). Although this result is not new the example illustrates how and that this approach works. More details about this analysis can be found in [15]. Such an analysis also enables the researcher to obtain detailed information, e.g. on the prevalence of risk factors in subgroups of the population. Thus, in the Bremen survey HEALTH! we also identified unexpected risk groups who might be of interest for further targeted intervention measures. For instance, we identified divorced women to show a high propensity to smoke, to become obese and to be less physically active. The decision whether e.g. such high prevalence groups should then be considered as target groups for specific interventions depends on further criteria (see above).

Both approaches are obviously based on available information: the literature-based approach needs that evidence for certain target groups can be obtained from review articles or original publications describing studies that have already been conducted in the research field of interest. Thus, literature reviews are not helpful in case of insufficient study designs in the past or new research questions. That is, new and innovative ideas about potential target groups to solve a public health problem will typically not result from a literature review but can be obtained from the second approach that we presented. This approach, however, has two major limitations. First, original data obtained from a population-based survey have to be available. However, this limitation may become less serious in the future as population-based health surveys are performed on a regular basis in various countries. Second, the identified target groups are obtained from an exploratory statistical analysis with all known caveats. We especially have to face the problem that the results obtained from exploratory techniques applied to high-dimensional data sets might not be stable. Thus, some kind of sensitivity analysis might be reasonable such as drawing bootstrap samples and performing the analysis repeatedly. In any case the obtained results are exploratory and they have therefore to be critically reflected regarding their reliability and validity. We recommend to combine both approaches whenever possible on the one hand to not miss any new ideas about potential target groups and on the other hand to have the opportunity to confirm the statistical results by the literature.

### Identification of modifiable risk factors and endpoints

After having identified appropriate target groups the second step on our roadmap to primary prevention deals with the specification of those variables that should be modified by the intervention programme to achieve a change in relevant endpoints that of course have also to be specified in advance. Since the study design also depends on the endpoints to be measured this section discusses not only how to identify potential intervention targets and outcomes but also relevant study designs.

The identification of the target group is closely related to the selection of the intervention targets. They define the endpoints which are of special importance regarding outcome evaluation and eventually the selection of the study design. To make this clear let us come back to the example how to combat the obesity epidemic. We already identified children in the age group from 2 to 10 as our target group to be recruited via the settings kindergarten and school. We know from our literature review that - besides eating behaviour and physical activity - stress is a modifiable risk factor of obesity [21]. This knowledge can be used to define specific intervention targets to be addressed by the planned intervention study.

We also learned from our literature review that intervention targets should address only one aspect, should be simple and positive resulting in few intervention messages such as drink more water (nutrition), provide safe bicycle lanes (physical activity) and share your worries (stress). The various intervention targets differ by the intervention level they address. "Drink more water" clearly addresses the individual, but also indirectly the family and the setting, since water has to be provided by both of them. "Provide safe bicycle lanes" is obviously targeting the community and "share your worries" the individual and the family.

#### Outcome measures

The outcome measures have to be determined with the evaluation of structure, process and effect in mind (see below). For the outcome evaluation it is best to use an endpoint that can be quantified. In the IDEFICS study we decided to measure the reduction of the prevalence of overweight and obesity in the intervention group compared to the control group to assess the effect of our intervention programme. We chose the body mass index (BMI) as the main endpoint because its components can be easily measured and it can be easily compared with published data. In addition, it correlates well with the amount of adipose tissue and it is a widely used marker of obesity with accepted reference values for children [22]. The effect measure has to be determined in the early planning phase of the study since it enables to fix the statistical study plan regarding power calculations and sample size determinations [15].

#### Study design

The selected outcome measures have an impact on the study design that has to fulfil certain requirements to make the assessment of the above measures possible. For instance, the study has to be longitudinal comparing a survey at baseline (T0) with one or more surveys at follow-up (T1, T2) after a pre-defined intervention period. By this design the sustainability of the programme can be investigated and the effect of the intervention with regard to a reduction of the prevalence of overweight and obesity over time can be measured.

This is, however, not sufficient to be sure about the effect of the intervention. The study also has to be a controlled trial where a pure design such a randomised controlled trial would be preferable but often not feasible due to practical limitations (see section on evaluation of structure, process and outcome). An alternative approach to an allocation of an intervention to individuals is a so-called cluster-randomization, especially in health services research. Such an approach is recommended if (1) the intervention is naturally applicable to clusters such as schools, hospitals or nursery homes, (2) there is the danger of contamination, e.g. by peers, if individual allocation is used and (3) a cluster-randomization is much less costly or more practical than an individual allocation. For more details we refer to e.g. Wears [23]. But please note that cluster-randomized trials do not apply to the first implementation of a programme because e.g. nothing is known about the intracluster correlation coefficient. This has to be known or at least reasonable assumption about its size should be possible, among others, to perform an adequate sample size calculation. Quasi-experimental designs, i.e. studies that lack random allocation of treatments, are a useful alternative as long as the drawbacks of such designs are taken into account. Their statistical analysis has to carefully account for confounders as it is the case in observational studies. They reduce the time and resources otherwise needed for randomisation. Finally, for most studies it is recommended to combine quantitative and qualitative approaches in the design. For examples we refer to the two subsequent sections.

Further aspects to be considered in the design are the selection of an appropriate outcome measure (see preceding and subsequent sections), treatment integrity, attrition and comparability of intervention and control groups. Treatment integrity, i.e. the degree to which an intervention is implemented as intended, needs to be high because interpretation of the obtained results requires assurance that treatment was carried out the way it was designed. If treatment integrity is low, it will be difficult to assign the observed changes to the intervention measures. This aspect is also part of the conflict between the positivist and the hermeneutic view as described in the section on evaluation of process, structure and outcome. Already in the planning of a longitudinal study measures to reduce attrition have to be foreseen. It is important to motivate study subjects to contribute to a scientific study. For this purpose they have to be taught well about the relevance and potential public health impact of the study to elicit their motivation to make an important contribution to the well-being of people in general. Besides by media involvement and well-organised information material (e.g. to be used for the informed consent) this may be achieved by regular updates of study results presented on a webpage in an easy-to-read manner. Also the personal interests of study subjects who want to obtain an individual benefit from their participation should be considered, e.g. by reporting back any measurement results that are meaningful to them (e.g. because they have a therapeutic benefit) and by giving them tailored feedback and advice based on their data. Finally, the comparability of intervention and control groups requires special attention in quasi-experimental designs. Researchers should strive to equalise both groups as much as possible with regard to any variables that can be related to the outcome of interest. Means to achieve this are for instance a clear definition of inclusion criteria and a pre-interventional screening of e.g. comparison communities with regard to their socio-economic structure. Since these measures will, however, hardly ever ensure full comparability of both groups, the corresponding confounder variables need to be measured and taken into account in the statistical analysis. Moreover, it is recommended to perform a post hoc comparison of both groups regarding the distribution of potential confounders.

For the IDEFICS study it was therefore decided to compare two communities, an intervention and a control region, with a similar socio-demographic and socio-economic structure. These regions had to be distant enough to prevent contamination of the control region by intervention activities. In each intervention and non-intervention region we recruited about 500 pre-school children being 2 to 4 years old at baseline and 500 school children being 6 to 8 years at baseline. Thus, about 2.000 children were recruited in eight countries each, leading to about 16.000 children in total. This sample size also allows to show statistically significant differences of small size (Cohen's d = 2) with a power of 80% for subgroups on country level or for girls/boys in kindergartens and schools [24].

### Development and implementation of an intervention programme

In the following, we highlight the importance of a theoretical model as basis of each intervention programme and the importance of a participatory approach involving all relevant actors in the development and implementation phase.

The development and implementation of an intervention programme has to account for the socio-cultural and living environment of the target groups. Furthermore it should follow a participatory approach, e.g. to assess the needs and problems of target groups or local people [25]. Target groups may be involved to different degrees: information sharing, consultation, collaboration, full responsibility [26-28]. Although this implies that sufficient start-up time has to be considered when planning the overall time frame, this may help to increase the acceptance and sustainability of the final programme.

In the IDEFICS study, for instance, focus groups were conducted with teachers, parents and school children. Especially, families with migration background and low SES were involved to learn how to reach those groups. Topics of the focus groups addressed diet, physical activity and stress. The intention was to identify needs and barriers but also supporting factors for a healthy lifestyle at various levels, i.e. community, kindergarten and school as well as family [29]. The information we gained were then used for the development of the intervention modules as well as for their implementation. As an example, it was found from the parental focus groups that insufficient space for active breaks at school was commonly perceived by the parents as a major problem in most of the countries. On the basis of this observation, an intervention module was developed aimed at supporting school boards in increasing the "play-ability" of the schoolyards with or without spatial enlargements.

The overall process of intervention development was guided by the intervention mapping approach as a general framework [30] which consists of the following steps: (1) definition of proximal programme objectives which include the performance objectives and their determinants, the identification of target groups and the learning objectives, (2) selection of suitable theoretical methods and practical strategies, (3) design of the programme plan, (4) adoption and implementation plan and (5) monitoring and evaluation plan.

Intervention measures that are theory-driven and that have shown empirical evidence are the most promising ones. Because of the various levels and the complexity of the setting approach we cannot rely on only one theory but have to take several theories into account. For instance, elements of health education combined with knowledge transfer may be used at the family level where the transtheoretical model [31] may form the theoretical framework to change parental behaviour. In addition, we will draw upon existing modules for which evidence is at least promising. The modules should be standardized to make reproducibility of results possible and to allow for comparisons in a multi-centre study such as the European IDEFICS study. But note that even if the intervention modules have been developed in a standardized way they have of course to be culturally adapted.

It should also be kept in mind that the interveners that are involved in the practical implementation of modules may induce effects that are beyond the ones originally intended by the module's concept. It requires special attention and care to make sure that interveners not only adhere to the protocol but also present with the right dose in their commitment such that modules are deployed in a way that offers the highest guarantee for the desired effect. In order to achieve this, detailed protocols together with targeted training are often required. In addition, it is in most instances of interest to create a forum where interveners can exchange experiences amongst themselves, get support for troubleshooting and learn from each other. Last but not least, instruments should be developed in most cases that allow for the objective measurement of compliance and accuracy among interveners in such a way that remediation can be done whenever required.

The various modules should be easy to implement in the daily routine of a setting like a kindergarten without requiring special facilities or instruments. The intervention modules have to be of low cost to enable their wide-spread use. As mentioned before specific modules are needed to reach the defined targets at all intended levels and to also reach vulnerable groups.

After being developed the intervention modules should be implemented in a non-directive and participatory way. The implementation procedure may be worked out in close collaboration with all actors involved to make the programme acceptable and sustainable. This may be achieved by establishing a round table with all actors, local key players and stakeholders. The focus groups also confirmed that target groups may best be reached through the settings in which they are integrated while acceptability is improved if the intervention programme is connected with local networks such as associations of migrants or boards of school principals. All activities should be coordinated through one project leader or management group that acts at the local level and that is made well known to all actors such that they know whom to talk with if problems occur or if details of a specific event have to be worked out.

It is of utmost importance that an intervention programme is known to the public when its implementation begins. For this purpose various media like lay-press and local broadcasting stations should be approached to promote the intervention messages already at an early stage. A detailed public relation strategy can support the corresponding media campaign at all stages of the project.

### Evaluation of structure, process and outcome

This section deals with the evaluation of primary prevention programmes, which is a challenge for epidemiology and public health sciences, where we try to find a compromise between a positivist and a hermeneutic view. On the one hand we are confronted with the requirements derived from evidence-based medicine (EBM) that should also be met by primary prevention programmes and that give highest priority to the results obtained from randomized controlled trials. On the other hand - this has been shown by numerous publications in the context of public health - we face conditions in the framework of public health interventions that do not allow an unrestricted transfer of the EBM paradigm [e.g. [32-35]].

Koelen et al. [36] distinguish three dilemmas of the biomedical research paradigm:

1. The intervention and outcome dilemma addresses the problem that it is often not possible to specify the independent variable, i.e. the intervention, in advance. The reasoning behind is that we do not only want to change individual behaviour but also to alter the social, cultural and organisational environment. To achieve a sustainable change it is necessary to involve the target groups and the stakeholders. This typically implies a dynamic change of the intervention in the course of the implementation process that cannot be foreseen in the planning stage.

2. The number dilemma addresses the problem that research approaches in biomedicine require quantitative measures, while the aims of public health interventions are often hardly measurable as e.g. the development of healthy lifestyles and the empowerment of people and communities.

3. The control group dilemma addresses the problem, as already described above, that randomization is usually impossible.

According to Koelen et al. [36] the challenge is now to reconsider (1) the role of research, (2) the definition of outcome variables, (3) the research methods and instruments, and (4) the criteria to judge validity.

This debate relates to the epistemological controversy between a positivist view and a hermeneutic view [as e.g. discussed by [37]]. The positivist view is among others characterized by objective observation, by aiming at explanation and prediction, by searching for general knowledge and standardization, by hypothesis testing through formal definition of ideas and measurements, by aiming at controlling the collection of facts with emphasis on quantity. This conflicts with the hermeneutic view characterized by critical subjectivity, by aiming at understanding and finding meaning, by viewing every situation as unique, by conducting a dialectical cycle to gain knowledge, by aiming at enlightenment, edification, enrichment and personal growth with emphasis on quality which may impair treatment integrity (see section on study design).

We seek for a combination of both views to reach the target of a sustainable and evidence-based intervention programme that is accepted and maintained by the target groups themselves after the research programme is finished [see also [38]]. It is our rationale to use and combine various research approaches and methodologies, as long as they are reasonable, to adequately evaluate the respective research topic.

A more hermeneutic example is given with the project "Quali-Set-Practice" that follows a participatory research design. It develops within a comprehensive health promotion programme for children and adolescents in a district of the city of Mainz. All residents in this district, in total about 300 at the beginning of the intervention in 2003, are socially deprived. The programme offers local health promotion activities, builds networks and initializes co-operations, exploits existing institutional and individual resources while taking gender and cultural differences into account. "Quali-Set-Practice" aims at analyzing the functional principles of health promotion and refers to the dilemmas described by Koelen et al. [36] in addressing the following questions:

(1) How does a health promotion programme work in the setting town district? How are activities for health promotion created and how do they develop? Which are the roles of the different actors (target groups, social workers, community members, sponsors) in the development of these activities?

(2) Which ideas do the various actors have about the aims of an evaluation? Which evaluation concepts are adequate for which activities? Do activities exist that are amenable to a statistical/epidemiological evaluation?

So far, results refer mainly to the first set of questions where reconstructive methods like analysis of documents and interviews with different actors (programme providers, representatives of the target group etc.) prevailed. Almost 100 different activities were identified. Some take place regularly and persistently like activities to promote physical exercise or relaxation while others are created for specific occasions like the planting of pumpkins for Halloween. These activities can be grouped according to different areas: healthy nutrition, physical activity, relaxation, vaccination, and health education. A specific activity may be assigned to various areas simultaneously or subsequently. It can also aim at different specific targets.

Let us illustrate this aspect using an activity related to physical exercise for children and adolescents. The original concept has foreseen to develop various activities for physical exercise targeting different groups without specifying them. The first specific activity - a soccer training for male adolescents - was developed on request of the male adults to keep the adolescents out of the streets. For the programme providers this was seen as an opportunity to get in closer touch with the whole target group. After its implementation, new targets emerged like the improvement of motor skills and social learning and the programme was further differentiated. The possibility to relax in a specially designed room with light effects and music, referred to as "snoezelen" http://www.isna.de[39], may serve as another example. This activity first aimed at providing nice relaxing experiences to children and at further developing the concept of snoezelen for children from socially deprived families as a specific target group. It now also aims at linking snoezelen with the improvement the reading skills. In principle, this last target is amenable to a stringent scientific evaluation.

An evaluation needs to be planned carefully [40,41]. Typically only specific aspects of a whole intervention programme can be assessed systematically and these have to be identified before starting the programme. It is necessary to specify the methods that should be used for evaluation. Depending on the methods appropriate assessment criteria have to be derived. These methods and criteria have to be taken into account already when planning an intervention programme. The choice for a specific approach partly depends on the person or institution that carries out the evaluation. This could be external scientists, contract research organizations, researchers being involved in the programme or the target group itself. An early choice avoids unclear responsibilities and guarantees a systematic evaluation according to scientific standards as requested e.g. by the DeGEval [42]. Finally, the whole evaluation process also depends on the objectives that are pursued by the evaluation. In our example of the IDEFICS study we have formulated the whole course of the study and thus also the evaluation process as a so-called logical framework that follows consecutive steps. For each step the according target is defined and it is specified, which information has to be obtained for evaluation purposes and by which criteria it has to be assessed.

According to Kromrey [40] these requirements have consequences for the study design. The planned study should allow to measure precisely each single measure of the programme (independent variables), to identify all circumstances that might influence the results of the study (exogenous variables), and to measure all effects, intended or not (dependent variables), which of course requires an appropriate operationalization of the effects to be investigated. It is necessary that all aims of the study are specified before the design of the study is developed. During the course of the study neither the aims nor the major circumstances may change to enable a stringent scientific evaluation. Hypotheses regarding the research aims should be theory-based and have to be formulated before the study design is fixed. The researcher needs to control the research process to ensure the internal and external validity of the results. Kromrey [40] advocates to use the classical field experiment as a reference design where an intervention group that is exposed to the treatment is compared with an unexposed control group. In both groups the dependent variables that we expect to change due to the intervention have to be measured before start of the intervention and after sufficient follow-up time. Given that both groups are comparable differences between the dependent variables are interpreted as effects of the intervention programme. Such a pure experimental design cannot always be applied. Alternative designs that are methodologically acceptable have then to be identified [40]. For instance, in the IDEFICS study we decided to measure potential confounders to enable their control in the statistical analysis instead of controlling them by randomization.

We hope that the IDEFICS study may turn out as a good practice example where quantitative methods such as a controlled design and statistical testing of effects and qualitative methods such as focus groups are combined to achieve the best evidence. In the IDEFICS study all measures will be evaluated regarding structure, process and outcome. In detail the evaluation addresses

• structure: assessment of costs of the various modules, assessment of time needed for implementing and running the modules, reporting of practical problems and development of potential solutions;

• process: qualitative interviews of parents, teachers and further actors with respect to compliance, perception, feasibility, acceptance and sustainability. These interviews will take place after 6 months and after termination of the intervention;

• outcome: comparison of anthropometric data before and after the intervention and in comparison to the control region. Besides e.g. the BMI indicators of energy balance and nutrition, indicators of stress coping, biomedical and psychosocial parameters will be investigated.

### Bringing an evidence-based intervention into practice

Let us finally discuss the probably most challenging step on our roadmap to primary prevention. After having developed an evidence-based intervention programme which has proven its effectiveness in a quasi-experimental setting the aim is to promote it and to make this programme available to the public. At a first glance this seems to be an easy task, since - in the researcher's view - such a programme has been awaited eagerly. However, this transfer is extremely time-consuming and laborious. It is beyond the scope of this paper to describe in full detail the transition from the scientifically examined concept to the fully operational practice. Some key considerations relate to the general strategy, the communication and the involvement of local actors.

To be honest, such a clear border where science ends and promotion and policy start does not exist in our view. The process of bringing a programme into practice has to start much earlier that is before its evidence is proven. At least from our experience, it is highly recommended to involve, where appropriate, politicians, educators, health professionals, parents and other relevant actors as soon as possible. As already mentioned in the section about development and implementation of an intervention programme, acceptance of a programme and thus also later use after having shown its effectiveness need a participatory approach. Thus, the border between scientific and promotion is rather fluent with respect to development, implementation and also with respect to the whole process of transfer although activities of bringing a programme into practice have to be strengthened after the effectiveness has been shown taking the following aspects into account.

As scientists we are used to present our research results on scientific conferences and workshops and to publish in peer-reviewed scientific journals to comply with the principles of good scientific practice and to foster scientific exchange. Since this is not sufficient to transfer the results gained from prevention research into practice, the German Association of Epidemiology recommends in its Guidelines for Good Epidemiological Practice: "Epidemiological studies that are concerned with the transfer of results into health-promoting measures should adequately involve the affected groups of the population and they should thrive for a qualified risk communication with the interested public" [translated from [43]].

A first step in this direction is to present the results and their consequences on meetings that primarily address the target groups and that are e.g. organized by and for the various groups of stakeholders involved. It is necessary to prepare easy-to-read articles in public press and to prepare press releases for daily newspapers and further media that reach a broader public. For this purpose support by a communication scientist may be helpful.

A context analysis - on an ad hoc basis - should reveal potential barriers that might require critical changes in some of the intervention modules or otherwise more intensified monitoring of the process. Some of the classical settings like schools can be fairly unpredictable in terms of reaction or "behaviour" vis-à-vis new procedures or tools.

A council consisting of representatives of various stakeholders and potential channels, especially integrating representatives of organisations and institutions devoted to health promotion should be established to discuss the results of the prevention programme. This may also increase the public awareness of the study results by asking the members of the council for their support to make the programme known via their networks. Such a council should be established in parallel to the ongoing prevention trial. This does not only accelerate the communication with the public, but also considers the perspectives of the various stakeholders in an early stage of the project for instance when formulating the key messages. This may help to counteract potential problems with acceptability of the intervention programme in a pro-active manner.

Of outmost importance is the careful recruitment and remuneration of the key interveners and facilitators of the intervention. Motivated, qualified and well-trained persons will make the difference between failure and success in all its gradations. However well designed an intervention programme may be, it will turn out the wrong way if it is not borne by interveners who are highly committed to the programme and highly skilled.

Guidelines and easy-to-understand manuals should be provided to the various interest groups and politicians that may help in decision-making processes. Furthermore, the provision of target-group specific manuals and instruments can promote the direct transfer of particular intervention modules into practice which should be complemented by train the trainer programmes.

All actions should be consolidated. For this purpose, the publication relation strategy that enhances visibility of the intervention programme should also foresee steps to go after completion of the project when the effectiveness of the programme hopefully has been shown.

## Summary

Starting from a given health problem suitable target groups have to be identified, modifiable risk factors have to be chosen and realistic targets of the intervention have to be specified. Target groups and outcome measures are the cornerstones on which the study design and the definition of comparison groups should be built. The development of the intervention programme has to take into account the socio-cultural and living environment and may follow participatory approaches which require sufficient start-up time. Evaluation is the key component of any intervention which should not only assess structures and processes but particularly evaluate the outcome and include the effectiveness of the programme according to rigid scientific criteria. One of the most challenging steps is the implementation of evidence-based intervention modules into practice and to achieve their sustainability.

## Competing interests

The authors declare that they have no competing interests.

## Authors' contributions

Conception and design: IP, SDH, IJ, WA. Acquisition of data: IJ. Analysis and interpretation of data: IP, RF, IJ, WA. Drafting and revising the manuscript: all authors. Final approval of the version to be published: all authors.

## Pre-publication history

The pre-publication history for this paper can be accessed here:

http://www.biomedcentral.com/1471-2288/10/10/prepub
